# Bacteriology and drug susceptibility analysis of pus from patients with severe intra-abdominal infection induced by abdominal trauma

**DOI:** 10.3892/etm.2014.1609

**Published:** 2014-03-06

**Authors:** SHAOYI ZHANG, LELE REN, YOUSHENG LI, JIAN WANG, WENKUI YU, NING LI, JIESHOU LI

**Affiliations:** Department of Surgery, Jinling Hospital, Nanjing University School of Medicine, Nanjing, Jiangsu 210002, P.R. China

**Keywords:** abdominal trauma, severe intra-abdominal infection, pus, bacteriology

## Abstract

The aim of the present study was to retrospectively analyze the bacteriology and drug susceptibility of pus flora from abdominal trauma patients with severe intra-abdominal infection (SIAI). A total of 41 patients with SIAI induced by abdominal trauma were enrolled in the study, from which 123 abdominal pus samples were obtained. The results from laboratory microbiology and drug sensitivity were subjected to susceptibility analysis using WHONET software. A total of 297 strains were isolated in which Gram-negative bacteria, Gram-positive bacteria and fungi accounted for 53.5 (159/297), 44.1 (131/297) and 0.7% (2/297), respectively. Anaerobic bacteria accounted for 1.7%. The five predominant bacteria were *Escherichia coli* (*E. coli*), *Staphylococcus aureus* (*S. aureus*), *Klebsiella pneumoniae* (*K. pneumoniae*), *Enterococcus faecalis* and *Pseudomonas aeruginosa* (*P. aeruginosa*). *E. coli* was highly susceptible to cefoperazone (91%) and imipenem (98%), while Gram-positive cocci were highly susceptible to teicoplanin (100%) and linezolid (100%). *S. aureus* was 100% susceptible to vancomycin and *K. pneumoniae* was highly susceptible to imipenem (100%) and amikacin (79%). *P. aeruginosa* was the most susceptible to ciprofloxacin (90%). Gram-negative bacterial infection was present in the majority of cases of SIAI. However, a large number of patients were infected by Gram-positive bacteria, particularly *S. aureus* that exhibited significant resistance to penicillin (100%), oxacillin (100%) and a third-generation cephalosporin antibiotic cefotaxime (95%). Amongst the pathogenic bacteria that cause SIAI, both Gram-negative and Gram-positive bacteria account for a high proportion, so high-level and broad-spectrum antibiotics should be initially used.

## Introduction

Infections have long been known to complicate care in patients after traumatic injury frequently leading to excess morbidity and mortality (1). Intra-abdominal infection is the most common infection in abdominal trauma patients with an incidence rate of 2–9% (2,3). It is frequently complicated with acute respiratory distress syndrome, multiple organ failure, gastrointestinal fistula, abdominal wall defects and malnutrition (4). Uncomplicated intra-abdominal infections, including suppurative appendicitis, can be eliminated simply by surgery with prophylactic anti-infective drugs. By contrast, patients with severe intra-abdominal infection (SIAI) that is persistent and complicated with progressive organ dysfunction require the administration of antibiotics in addition to surgical intervention.

SIAI refers to intra-abdominal infections complicated by sepsis and septic shock (5). The mortality rate of SIAI can reach 50% (6) and antibiotic intervention is required upon onset. Bacteriological and susceptibility analyses of pus and whole blood may aid the selection of antibiotics in treating abdominal infection, prior to which antibiotics should be empirically administered. At present, antibiotics are empirically administered to SIAI patients based on the worldwide Study for Monitoring Antimicrobial Resistance Trends (SMART) and the domestic CHINET bacterial resistance surveillance (7,8). However, pus derived from the deep abdominal cavity has seldom been subjected to bacteriology and susceptibility analysis. Therefore, the present study retrospectively analyzed the spectrum of bacterial infection and drug resistance changes of pus in patients with intra-abdominal trauma and SIAI who were admitted to Jinling Hospital, Nanjing University School of Medicine (Nanjing, China) between January 2001 and May 2012. The aim of the study was to increase the accuracy of empirical medication. Considering the poor survival rates of SIAI patients and the lack of studies investigating the bacterial cultures of abdominal cavity pus, the results of the present study are particularly significant for clinical practice.

## Materials and methods

### General information

A total of 274 intra-abdominal trauma patients (age, 38.2±19.7 years) who had been enrolled in Nanjing General Hospital between January 2001 and May 2012 were selected for this study, including 225 males and 49 females. There were 196 cases of closed injury and 88 cases of open injury, including 176 traffic accidents, 50 fall injuries, 32 collision injuries and 16 sharp injury cases. Patients were treated between 0.5 and 24 h following the trauma. A total of 191 patients were admitted to the emergency room immediately following trauma, of which 48 cases were complicated with shock. A total of 41 out of the 96 intra-abdominal infection cases suffered from SIAI. The detailed injury statuses are summarized in Table I. This clinical study is approved by the ethic committee of the Jinling Hospital Nanjing University. The collection of all the clinical specimen are under the authorization of the patients or the patients’ families.

### Screening of subjects

**Figure 1:**
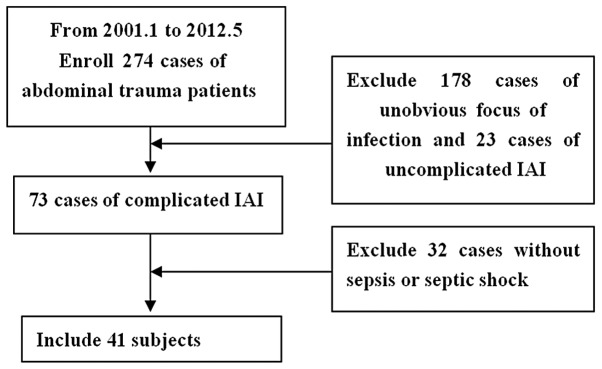
Screening of subjects. IAI, intra-abdominal infection.

Patients were diagnosed with SIAI if the intra-abdominal infections were complicated with sepsis and/or septic shock (Fig. 1).

Systemic inflammatory response syndrome (SIRS) was diagnosed if patients had two or more of the following symptoms: i) a body temperature of >38°C or <36°C; ii) a heart rate >90 bpm; iii) a respiratory rate >20 breaths/min or a PaCO_2_ value <4.26 kPa (32 mmHg); and iv) a white blood cell count of >12×10^9^/l or <4×10^9^/l or a stab granulocyte count of >0.10. The diagnostic criteria of sepsis were in accordance with those for SIRS with definite evidence of infection (9).

Septic shock was diagnosed in patients with a systolic blood pressure of <90 mmHg or whose systolic blood pressure had decreased by ≥40 mmHg on the basis of the original value, with or without symptoms associated with poor tissue perfusion, including acidosis, oliguria or acute consciousness disorders (10).

### Sample collection

All samples were collected from the patients during surgery or in intensive care. From the deep abdominal cavity, ≥1 ml pus (gallbladder bile and bile in the gallbladder wall or common bile duct were not included) was collected using a disposable sterile syringe, which was quickly sealed in a sampling tube. The samples were sent to a laboratory within 2 h for aerobic and anaerobic cultures.

### Pathogenic examination and susceptibility determination

Abdominal pus samples were routinely cultured in a BACTEC 9120 automated blood culture system (BD Diagnostics, Sparks, MS, USA) which raised an alarm when cases testing positive for bacteria were identified. Samples yielding positive results were subjected to susceptibility tests using the Kirby-Bauer disk diffusion susceptibility method, according to the National Committee for Clinical Laboratory Standards (2011) (11). Diameters of the zones of complete inhibition (as judged by the unaided eye), including the diameter of the disk, were measured. Zone margins were considered as the area exhibiting no marked or visible growth that is was possible to detect by the unaided eye. The results of the susceptibility tests were reported as susceptible, intermediate or resistant. Gram-positive and -negative bacteria were identified using the Vitek-32 microbial identification system and analytical profile index strips purchased from BioMérieux (Lyon, France). Control strains, including standard *Staphylococcus aureus* (*S. aureus*; ATCC25923), *Escherichia coli* (*E. coli*; ATCC25922) and *Pseudomonas aeruginosa* (*P. aeruginosa*; ATCC27853), were provided by the Quality Control Center of Jiangsu Province (Lianyungang, China). WHONET 5.4 software developed by WHO Collaborating Centre for Surveillance of Antimicrobial Resistance based at the Brigham and Women’s Hospital in Boston was used to analyze laboratory findings.

## Results

### Types and distribution of pathogenic bacteria

From the 41 SIAI patients, 123 positive pus samples were collected (100%) from which 297 strains were isolated, including 131 strains of Gram-positive bacteria (44.1%) and 159 strains of Gram-negative bacteria (53.5%). In addition, 5 strains of anaerobic bacteria (1.7%) and 2 strains of fungi (0.6%) were isolated. *E. coli*, *S. aureus* and *Klebsiella pneumoniae* (*K. pneumoniae*) were the most predominant bacteria. The flora distribution is shown in Table II.

### Susceptibility of pathogenic bacteria

Pathogenic bacteria are prone to resistance against a number of antibiotics. Gram-negative bacteria exhibited the highest susceptibility to imipenem, but were resistant to cephalosporins. *E. coli* was highly susceptible to cefoperazone (91%) and imipenem (98%), while *K. pneumoniae* was highly susceptible to imipenem (100%) and amikacin (79%). However, >67% of *P. aeruginosa* strains tolerated imipenem and were treated most effectively by a quinolone antibiotic ciprofloxacin (90%). Gram-positive cocci, which were generally not susceptible to cephalosporins, exhibited 100% susceptibility to teicoplanin and linezolid. *S. aureus* was susceptible to vancomycin (100%) and *Enterococcus faecalis* (*E. faecalis*) was particularly susceptible to teicoplanin and linezolid without drug-resistant strains (Table III).

## Discussion

Currently, SIAI patients are empirically administered antibiotics at an early stage, based on international SMART research and the domestic CHINET bacterial resistance surveillance on collected samples from the respiratory system (46.9%, e.g. sputum), urine (19.9%), blood (11.9%), pus (5.2%), sterile body fluids (4.0%), genital tract secretions (1.7%), feces (1.2%) and others (8.2%) (7). To date, studies on the bacterial culture and drug resistance of pus in the deep abdominal cavity remain scarce. In the present study, Gram-negative bacteria (53.5%) primarily contributed to SIAI, in which *E. coli* (24.2%) and *K. pneumoniae* (11.4%) predominated. The results are consistent with those of a previous SMART study that analyzed patients from 14 centers in six countries in the Asia-Pacific region (12), in which Gram-negative enterobacteria accounted for 82% of cases of intra-abdominal infection (*E. coli*, 43%; *K. pneumoniae*, 20%). In addition, the resistance rate of *E. coli* to ceftazidime was only 17.5% in the aforementioned study, but this was elevated to 58% in the present study. The susceptibilities of *E. coli* to imipenem in the two studies exceeded 98%. Therefore, the analysis and review of the local bacteria distribution and susceptibility results of abdominal pus is crucial.

Intra-abdominal infection, which refers to an infection of an organ in the abdominal cavity, with the exception of peritonitis, may be divided into uncomplicated and complicated infections (13). Uncomplicated intra-abdominal infections are infections of only one organ with intact anatomical structure. By contrast, complicated infections, which are intrinsically secondary intra-abdominal infections, represent intra-abdominal abscesses or peritonitis following the invasion of pathogenic bacteria into the abdominal cavity from involved organs. Complicated intra-abdominal infections are often associated with intra-abdominal visceral perforation, ischemic gangrene and penetrating injury. It is possible to recover the majority of uncomplicated intra-abdominal infections by surgery without conventional antibiotic treatment, with the exception of prophylactic antibiotics. However, complicated intra-abdominal infections require treatment combining surgical protocols with anti-infective agents.

SIAI, as a complicated intra-abdominal infection, mainly manifests as diffuse peritonitis or multiple intra-abdominal and peritoneal abscesses, including severe pancreatitis, hollow organ perforation and anastomotic fistula. SIAI is commonly accompanied by apparent sepsis and intra-abdominal infection due to the invasion of numerous bacteria and toxins into the blood circulation, which can thus be referred to as sepsis of abdominal origin (incidence rate, 10%) (14). In addition, patients with SIAI are extremely vulnerable to acute respiratory distress syndrome and acute renal failure. In the present study, 39 out of 41 SIAI patients underwent continuous renal replacement therapy (95.1%) and 33 cases received tracheotomy for ventilator-assisted respiration (80.4%). Encountering uncontrollable infection sources, patients may succumb to constant or recurrent septic shock owing to the continuous release of bacteria and toxins into the blood. Thus, 50–70% of patients eventually succumb to multiple organ failure following respiratory and renal functional damage, as well as successive intestinal and hepatic dysfunction (15). A retrospective cohort study investigating secondary intra-abdominal infections verified that inappropriate initial treatment is likely to result in the failure of clinical treatment for SIAI, thus affecting the prognosis adversely (16). Notably, it is improper to excessively administer antibiotics at the outset of treatment.

**Figure 2:**
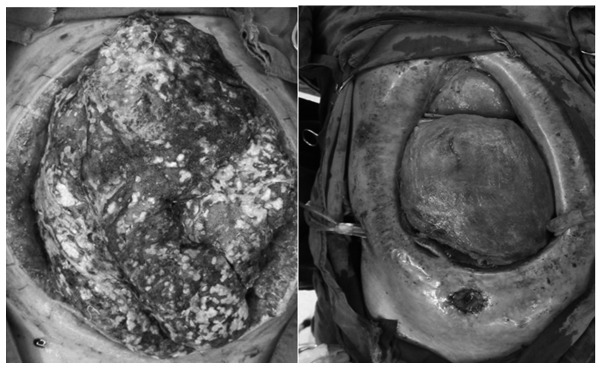
An SIAI patient undergoing multiple open abdominal surgeries following a car accident. SIAI, severe intra-abdominal infection.

Considering the abrupt onset, low survival rate and disunified antibiotic intervention of SIAI, as well as the lack of relevant bacteriology and drug resistance studies, in the present study the Department of General Surgery, as a national trauma rescue center, successfully intervened in SIAI patient treatment by culturing, analyzing and identifying associated bacteria. In the present study, *E. coli* and *K. pneumoniae* in patients with SIAI moderately tolerated cephalosporins by producing extended-spectrum β-lactamases (17). In addition, a small number of *K. pneumoniae* strains yield highly productive AmpC β-lactamase enzymes (18), which renders them highly resistant to third-generation cephalosporins that have been widely applied in clinical practice. Furthermore, undesirable inducible enzymes may be generated by *Enterobacter*, *Citrobacter*, *Serratia* and *Morganella* bacteria due to incautious administration of third-generation cephalosporins (19). The present study demonstrates that *E. coli* (98%) and *K. pneumoniae* (100%) were highly susceptible to imipenem, allowing this antibiotic to be administered with priority in the treatment of Gram-negative bacterial infection. In addition, the wide application of the third-generation cephalosporin brings is the increasing trend of the Gram-positive bacteria. In the present study, the significantly greater incidence of Gram-positive bacteria (44.1%) compared with that observed in the CHINET bacterial drug resistance surveillance in 2010 (28.4%) (7) may be associated with the high proportion of open abdominal cavities (36/41, 87.8%). In addition, a higher number of patients in the present study underwent abdominal surgeries. For example, one patient successively underwent five peritoneal irrigation and drainage procedures, as well as open abdominal surgeries, two abdominal gauze packing and removal surgeries and one enterostomy (Fig. 2). The resultant 18 strains of bacteria isolated from nine pus samples consisted of five strains of *E. coli* (27.8%), four strains of *S. aureus* (22.2%), four strains of *E. faecalis* (22.2%), three strains of *K. pneumoniae* (16.7%), one strain of *P. aeruginosa* (5.6%) and one of *Acinetobacter baumannii* (5.6%). Frequent open abdominal surgeries may directly increase the number of Gram-positive cocci in the pus culture.

Recently, third-generation cephalosporins have been combined with ornidazole, due to the coexistence of anaerobic and aerobic bacteria in severe or complicated intra-abdominal infections. These pathogenic bacteria may become highly resistant to common antibiotics, triggering refractory or secondary infections. With regard to previous studies, summarizing local bacteriology and susceptibility results provides clinical guidance for dealing with drug-resistant bacteria worldwide.

In summary, initial empirical antibiotic therapy should be modified based on susceptibility analysis results. In addition, patients with SIAI should be administered the most potent antibiotics immediately rather than the most commonly used antibiotics. Finally, it is critical to remove the sources of infection and to prevent intraoperative and postoperative bacterial contaminations in order to improve the therapeutic effects of eligible antibiotics.

## Figures and Tables

**Table I:** Statuses of the 274 abdominal injury patients.

Injury type	Cases, n
Simple abdominal wall injury	31
Abdominal viscera and retroperitoneal injuries^a^	219
Hepatorrhexis	49
Splenic rupture	77
Renal contusion	23
Pancreatic injury	68
Gastric and duodenal injuries	53
Small intestine rupture	24
Colon rupture	10
Complicated brain, chest, bone and urinary system injuries	24

a Single organ injury, 165 cases; multiple organ injury, 54 cases.

**Table II:** Distribution of 297 pathogenic microbial strains.

Type of pathogenic microbe	Strains (n)	Proportion (%)
Gram-positive bacteria	131	44.1
*Staphylococcus aureus*	66	22.2
*Enterococcus faecalis*	28	9.4
*Enterococcus faecium*	19	6.4
*Staphylococcus epidermidis*	12	4.0
*Staphylococcus haemolyticus*	6	2.0
Gram-negative bacteria	159	53.5
*Escherichia coli*	72	24.2
*Klebsiella pneumoniae*	34	11.4
*Pseudomonas aeruginosa*	21	7.1
*Stenotrophomonas maltophilia*	8	2.7
*Enterobacter cloacae*	5	1.7
*Chryseobacterium indologenes*	3	1.0
*Burkholderia cepacia*	3	1.0
*Acinetobacter calcoaceticus*	2	0.7
*Pneumobacillus*	2	0.7
*Chryseobacterium meningosepticum*	1	0.3
*Acinetobacter lwoffii*	1	0.3
*Citrobacter freundii*	1	0.3
*Morganella morganii*	1	0.3
*Pseudomonas cepacia*	1	0.3
*Proteus vulgaris*	1	0.3
*Alcaligenes xylosoxidans*	1	0.3
*Comamonas acidovorans*	1	0.3
*Acinetobacter baumannii*	1	0.3
Anaerobic bacteria	5	1.7
*Bacteroides fragilis*	1	0.3
*Bacteroides ovatus*	1	0.3
*Bacteroides thetaiotaomicron*	1	0.3
*Bacteroides distasonis*	1	0.3
*Bacteroides vulgatus*	1	0.3
Fungi	2	0.6
*Saccharomycetes*	1	0.3
*Candida albicans*	1	0.3

**Table III:** Percentages of R, I and S strains of five main pathogenic bacteria to common antibiotics.

	*E. coli* (72 strains), %			*S. aureus* (66 strains), %			*K. pneumoniae* (34 strains), %			*E. faecalis* (28 strains), %			*P. aeruginosa* (21 strains), %		
					
Antibiotics	R	I	S	R	I	S	R	I	S	R	I	S	R	I	S
Amikacin	11	8	81	22	5	73	12	9	79	64	4	32	19	5	76
Gentamicin	60	2	38	33	5	62	26	6	68	75	7	18	24	14	62
Ampicillin	49	22	29	39	6	55	100	0	0	43	7	50	100	0	0
Piperacillin	83	17	0	91	0	9	100	0	0	-	-	-	86	14	0
Cefazolin	61	7	32	36	3	61	100	0	0	-	-	-	-	-	-
Cefuroxime	61	3	36	28	5	67	100	0	0	-	-	-	-	-	-
Cefotaxime	58	3	39	95	5	0	32	6	62	-	-	-	48	38	14
Ceftazidime	58	6	36	-	-	-	29	6	65	-	-	-	19	19	62
Cefoperazone	4	5	91	-	-	-	24	3	73	-	-	-	90	10	0
Aztreonam	-	-	-	-	-	-	-	-	-	-	-	-	90	10	0
Imipenem	1	1	98	-	-	-	0	0	100	-	-	-	67	5	28
Ciprofloxacin	58	11	31	94	6	0	23	12	65	57	18	25	5	5	90
Piperacillin	83	6	11	-	-	-	12	15	73	-	-	-	24	0	76
Paediatric compound sulfamethoxazole	-	-	-	67	6	27	-	-	-	-	-	-	81	19	0
Vancomycin	-	-	-	0	0	100	-	-	-	0	14	86	-	-	-
Erythromycin	-	-	-	85	5	10	-	-	-	29	7	64	-	-	-
Penicillin	-	-	-	100	0	0	-	-	-	-	-	-	-	-	-
Oxacillin	-	-	-	100	0	0	-	-	-	-	-	-	-	-	-
Clindamycin	-	-	-	85	6	9	-	-	-	-	-	-	-	-	-
Phosphonomycin	-	-	-	36	0	64	-	-	-	-	-	-	-	-	-
Teicoplanin	-	-	-	0	0	100	-	-	-	0	0	100	-	-	-
Linezolid	-	-	-	0	0	100	-	-	-	0	0	100	-	-	-

R, resistant; S, susceptible; I, intermediate; *E. coli*, *Escherichia coli; S. aureus*, *Staphylococcus aureus; K. pneumoniae*, *Klebsiella pneumoniae; E. faecalis*, *Enterococcus faecalis; P. aeruginosa*, *Pseudomonas aeruginosa*.
